# Effectiveness and Safety of Acupoint Catgut Embedding for the Treatment of Poststroke Constipation: A Systematic Review and Meta-Analysis

**DOI:** 10.1155/2022/8080297

**Published:** 2022-07-05

**Authors:** Mao Guo, Xie Le, Wang Qin-yu, Mao Ye, Zhou Sheng-qiang, Xie Yao, Wu Da-hua, Liu Bai-yan

**Affiliations:** ^1^Hunan University of Chinese Medicine, Changsha 410208, Hunan, China; ^2^Affiliated Hospital of Hunan Academy of Chinese Medicine, Changsha 410006, Hunan, China; ^3^The People's Hospital of Hunan Province, Changsha 410005, Hunan, China; ^4^Hunan Academy of Chinese Medicine, Changsha 410006, Hunan, China

## Abstract

**Objectives:**

Acupoint catgut embedding therapy has shown effectiveness in treating functional constipation; however, relevant, high-quality clinical evidence is scarce. This study aimed to systematically assess the effectiveness and safety of acupoint catgut embedding in treating poststroke constipation.

**Methods:**

Correlative randomized controlled trials were identified through a comprehensive literature search of PubMed, Cochrane Library/Cochrane Central Register of Controlled Trials, Web of Science, Embase, China National Knowledge Internet, Chinese Biomedical Literature Database, Wanfang, and VIP databases from inception until February 2022. Meta-analysis was performed using RevMan 5.3 software.

**Results:**

Fifteen trials involving 1084 patients were identified. The meta-analysis revealed that the acupoint catgut embedding group was significantly superior to the non-catgut embedding group with regard to the efficacy rate (RR = 1.27, 95% CI (1.19, 1.37), *P* < 0.05), the first defecation time (MD = −3.08, 95% CI (−4.53, −1.63), *P* < 0.05), the defecation sensation score (MD = −0.44, 95% CI (−0.61, −0.26), *P* < 0.05), the degree of difficulty in defecation (MD = −0.73, 95% CI (−1.10, −0.37), *P* < 0.05), the PAC-QOL scale score (MD = −10.06, 95% CI (−13.47, −6.64), *P* < 0.05), and the symptom integral (MD = −3.15, 95% CI (−3.60, −2.71), *P* < 0.05). However, there was no significant difference in the stool property score (MD = 0.06, 95% CI (−0.39, 0.50), *P* > 0.05) as well as the incidence of adverse reactions (RD = 0.01, 95% CI (−0.01, 0.03), *P* > 0.05) between the two groups.

**Conclusions:**

The results showed that acupoint catgut embedding is probably an effective and safe acupuncture treatment strategy for poststroke constipation. Nevertheless, more rigorously designed, standardized, large-sample, and multicenter randomized controlled designs are warranted to further verify the findings of this study.

## 1. Introduction

Stroke is the leading cause of death and disability in many parts of the world. Authoritative research shows that the overall burden of stroke remains high worldwide and it is predicted that stroke will continue to be among the top three causes of death in the world until 2040 [1, 2]. Moreover, many stroke survivors endure physical and mental damage caused by some complications for a long duration after an acute stroke, which seriously affects the quality of life and prognosis of patients [3]. Constipation is a common poststroke complication. Approximately, 30% to 60% of stroke patients develop constipation symptoms after the event, which are mostly related to neurological disorders, dependence, long-term hospitalization, and motor, cognitive, and communication disorders [4, 5]. Constipation markedly harms stroke patients as it can result in symptoms or diseases such as abdominal pain, bad breath, depression, and hemorrhoids. In addition, it can induce another stroke or other cerebrovascular events due to prolonged squatting and forced defecation, thereby endangering the patient's life.

Thus, maintaining smooth defecation following a stroke is critical for these patients' prognosis. Currently, the clinical treatment of poststroke constipation consists mainly of diet adjustment, drug therapy (laxatives, kinetic agents), enema, and surgery. However, some of these treatments are ineffective, some are rejected because of poor tolerability, and the majority have significant adverse effects. In addition, the recurrence rate of poststroke constipation is high [6, 7]. In supplementary and replacement therapies, acupoint catgut embedding therapy is based on the theory of acupuncture and moxibustion in traditional Chinese medicine and uses absorbable surgical sutures to produce lasting acupoint stimulation in the human body, especially for poststroke constipation [8]. At present, high-quality clinical evidence of the acupoint catgut embedding therapy for the treatment of poststroke constipation is limited and the sample size of most related clinical studies is inadequate. Moreover, the efficacy, safety, and reliability of the acupoint catgut embedding therapy need to be improved. At the same time, there is no systematic evaluation of this problem. In view of this situation, this study used systematic evaluation and meta-analysis methods to evaluate the effectiveness and safety of the acupoint catgut embedding therapy in the treatment of poststroke constipation in order to provide a more reliable reference for clinical practice.

## 2. Data and Methods

The protocol was prospectively registered with the International Prospective Register of Systematic Reviews (PROSPERO) database on 5 March, 2022, (registration number: CRD42022310504.) and the International Platform of Registered Systematic Review and Meta-Analysis Protocols (INPLASY) on 13 February, 2022, (registration number INPLASY202220041). Literature search, data extraction, and quality evaluation were performed independently by two reviewers using the databases mentioned above, and any disagreements were resolved by consensus or by consulting a third experienced reviewer.

### 2.1. Search Strategy

A comprehensive search was performed in PubMed, Cochrane Library/Cochrane Central Register of Controlled Trials, Web of Science, Embase, China National Knowledge Internet, Chinese Biomedical Literature Database, Wanfang, and VIP databases from inception until February 2022. The following keywords or free-text terms were used: (poststroke or after stroke or after apoplexy) and (constipation or difficult defecation) and acupoint catgut embedding and randomized controlled trial. There were no restrictions on countries, population characteristics, and language for the search process.

### 2.2. Inclusion and Exclusion Criteria

The inclusion criteria were as follows: (1) the trials had to be RCTs that aimed to evaluate the therapeutic effect of acupoint catgut embedding on constipation after a stroke; (2) the subjects were patients who had poststroke constipation diagnosed according to WHO criteria, not limited by gender and age; (3) the intervention groups received the acupoint catgut embedding therapy, while the control groups received other therapies such as acupuncture, oral drugs, sham catgut embedding therapy, and so on; (4) the observation indices included at least one of the following: efficacy rate, first defecation time, defecation sensation score, degree of difficulty in defecation, stool property score, PAC-QOL scale score [9], symptom integral, and adverse event; and (5) there was a complete and clear treatment course. The exclusion criteria were as follows: (1) literature published repeatedly or published by more than one person in the same study (only the latest and the most comprehensive one was retained) and (2) studies in which the required data were unavailable, or studies for which attempts to contact the author to obtain missing data were unsuccessful.

### 2.3. Data Extraction

The contents of the data extracted mainly included the author, the year of publication, the country, the intervention measures of the experimental group and the control group, the number of cases in the experimental group and the control group, the course of treatment, the randomization method, and the outcome indicators.

### 2.4. Literature Quality Assessment

The Cochrane risk of bias tool [10] was used to evaluate the quality of the eligible randomized controlled trials. The tool mainly evaluated the risk of bias from 6 areas: selection bias, implementation bias, measurement bias, follow-up bias, report bias, and other biases. Each index was judged by “low risk,” “unclear,” and “high risk,” and the risk of bias distribution map was drawn.

### 2.5. Statistical Analysis

Revman5.3 software was used to draw the distribution map of the risk of bias and for meta-analysis. The counting data were expressed by relative risk (RR) and its 95% confidence interval (CI). The measurement data were expressed by mean deviation (MD) and its 95% confidence interval (CI). When I^2^ ≤ 50% and *P* > 0.10, the fixed-effect model was used to combine the data. When I^2^ > 50% and *P* < 0.10, the random-effects model was used to combine the data. When there was a large heterogeneity, the sensitivity analysis was carried out using the one-by-one elimination method to explore the source of heterogeneity. When the number of articles included in each outcome index was in the range of 2 to 10 articles, the publication bias among the included studies was evaluated by the Egger test using Stata16.0 software. *P* > 0.05 represents no significant publication bias.

## 3. Results

### 3.1. Literature Search Results

**Figure 1:**
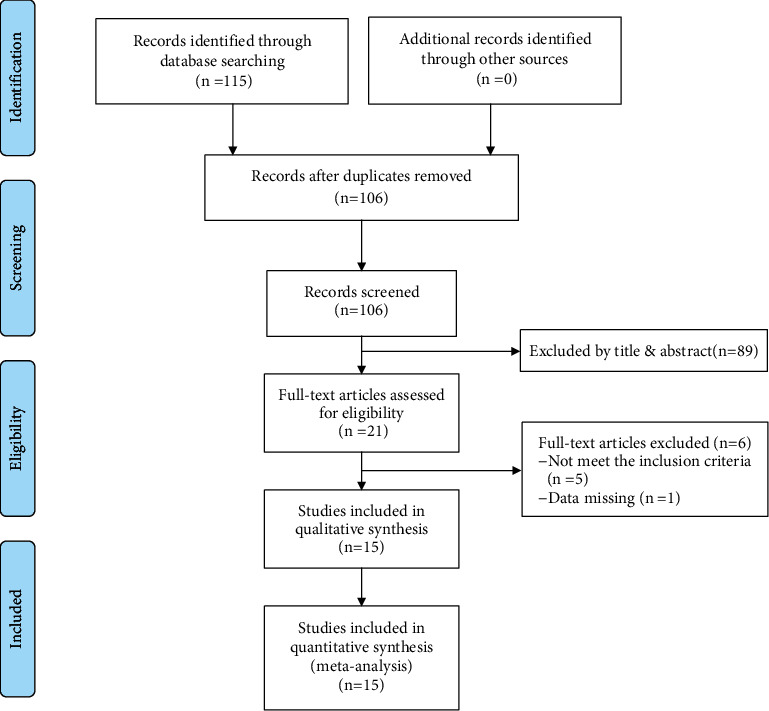
Flowchart of the literature selection process.

A total of 115 articles were initially selected from eight databases after preliminary screening. Then, the inconsistent studies were excluded based on their titles and abstracts and 21 articles were retained. Finally, the full texts of the remaining articles were evaluated, and the studies not meeting the inclusion criteria were excluded. Thus, 15 studies [11–25]were eligible for our systematic review. The specific search process and study selection are shown in Figure 1, and a detailed description of the general data is shown in Table 1.

### 3.2. Quality Assessment of the Included Trials

**Figure 2:**
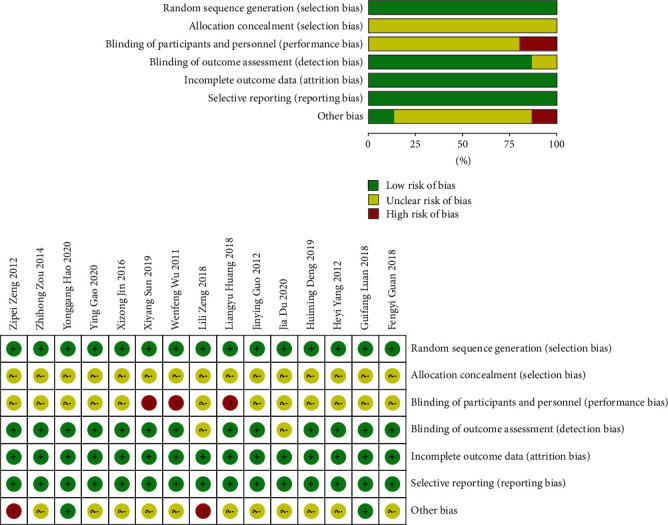
Literature quality risk bias chart.

We assessed the risk of bias in all the eligible articles. Randomization was mentioned in all the trials, including the following: 6 studies [12, 16, 20, 22–24] were randomized into groups by the random number table method, 2 studies [13, 14] were randomly divided into groups by statistical software, 2 studies [17, 21] was randomly divided according to the order of enrollment, and 5 articles [11, 15, 18, 19, 25] did not describe the specific method of randomization. Only two studies did not describe the blinding of outcome assessment. Methodological quality evaluation of the risk of bias is shown in Figure 2. The chart shows that there were many studies on low risk of bias, suggesting that the quality of the literature was acceptable.

### 3.3. Outcome Measures

#### 3.3.1. Efficacy Rate

**Figure 3:**
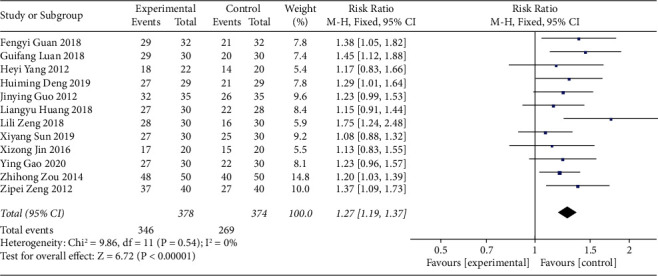
The forest plot of the efficacy rate.

Twelve studies reported the efficacy rate of acupoint catgut embedding for the treatment of poststroke constipation. The heterogeneity of the eligible studies was assessed, and the results (I^2^ = 0% and *P* > 0.10) indicated that there was no heterogeneity among the studies. Thus, the fixed-effects model was used to combine the data. The results revealed that the efficacy rate of the acupoint catgut embedding group was higher than that of the control group (RR = 1.27, 95% CI (1.19, 1.37), *P* < 0.05) (Figure 3).

#### 3.3.2. First Defecation Time

**Figure 4:**
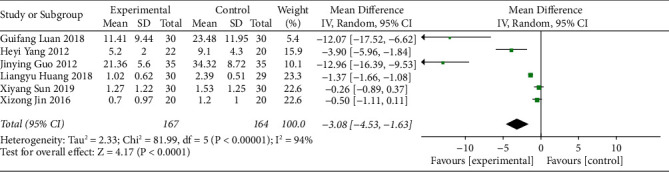
The forest plot of first defecation time.

Six studies reported the first defecation time of patients who received acupoint catgut embedding for the treatment of poststroke constipation. The heterogeneity of the studies was evaluated, and the results (I^2^ = 94% and *P* < 0.10) revealed a high degree of heterogeneity among the studies; therefore, the random-effects model was adopted. The results showed that the first defecation time of the acupoint catgut embedding group was shorter than that of the control group (MD = −3.08, 95% CI (−4.53, −1.63), *P* < 0.05) (Figure 4).

#### 3.3.3. Defecation Sensation Score [20]

**Figure 5:**
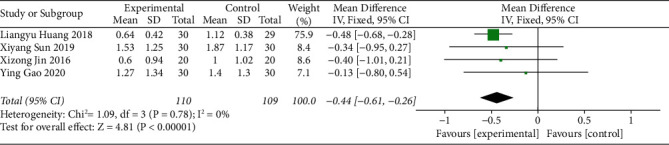
The forest plot of the defecation sensation score.

Four studies reported the defecation sensation score of acupoint catgut embedding receivers for the treatment of poststroke constipation. The heterogeneity of the eligible studies was tested, and the results (I^2^ = 0% and *P* > 0.10) showed that there was no heterogeneity among the studies. Hence, the fixed-effects model was applied to combine the data. The results demonstrated that the defecation sensation score of the acupoint catgut embedding group was lower than that of the control group (MD = −0.44, 95% CI (−0.61, −0.26), *P* < 0.05) (Figure 5).

#### 3.3.4. Degree of Difficulty in Defecation [14]

**Figure 6:**
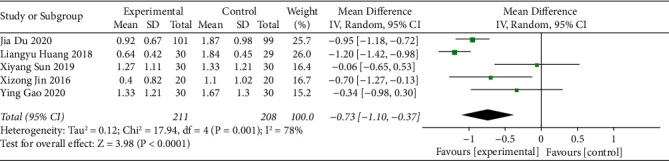
The forest plot of degree of difficulty in defecation.

Five studies reported the degree of difficulty in defecation for patients who received acupoint catgut embedding for the treatment of poststroke constipation and the heterogeneity of the studies was assessed. The results (I^2^ = 78% and *P* < 0.10) revealed a high degree of heterogeneity among the studies. Therefore, the random-effects model was used. The results indicated that the degree of difficulty in defecation of the acupoint catgut embedding group was lower than that of the control group (MD = -0.73, 95% CI (-1.10, -0.37), *P* < 0.05) (Figure 6).

#### 3.3.5. Stool Property Score [14]

**Figure 7:**
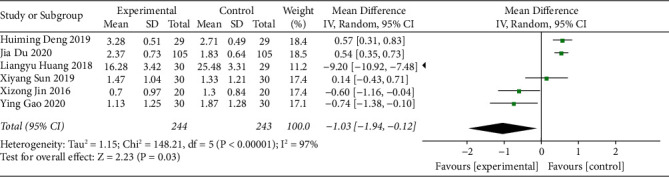
The forest plot of the stool property score.

Five studies reported the stool property score of acupoint catgut embedding receivers for the treatment of poststroke constipation. There was a high degree of heterogeneity among the studies (I^2^ = 86% and *P* < 0.10), and the random-effects model was applied. The results revealed that there was no significant difference in stool property scores between the two groups. (MD = 0.06, 95% CI (-0.39, 0.50), *P* > 0.05) (Figure 7).

#### 3.3.6. PAC-QOL Scale Score

**Figure 8:**
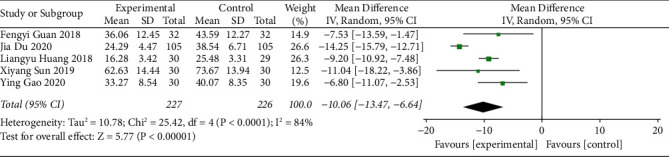
The forest plot of the PAC-QOL scale score.

The PAC-QOL scale score of acupoint catgut embedding receivers for the treatment of poststroke constipation was reported in five studies. The heterogeneity of the studies was determined. Since there was a high degree of heterogeneity among the studies (I^2^ = 84% and *P* < 0.10), the random-effects model was adopted. The results showed that the PAC-QOL scale score of the acupoint catgut embedding group was lower than that of the control group (MD = −10.06, 95% CI (−13.47, −6.64), *P* < 0.05) (Figure 8).

#### 3.3.7. Symptom Integral

**Figure 9:**
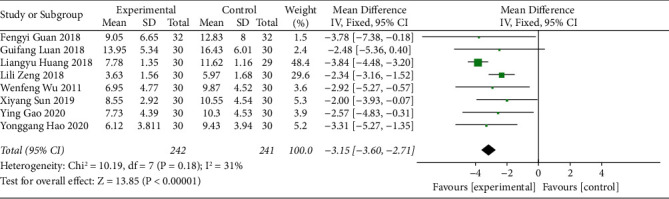
The forest plot of Symptom integral.

Eight studies reported the symptom integral of acupoint catgut embedding for the treatment of poststroke constipation. Heterogeneity assessment revealed that there was little heterogeneity among the studies (I^2^ = 31% and *P* > 0.10). Therefore, the fixed-effects model was used. The results demonstrated that the symptom integral of the acupoint catgut embedding group was lower than that of the control group (MD = -3.15, 95% CI (-3.60, -2.71), *P* < 0.05) (Figure 9).

#### 3.3.8. Adverse Events

**Figure 10:**
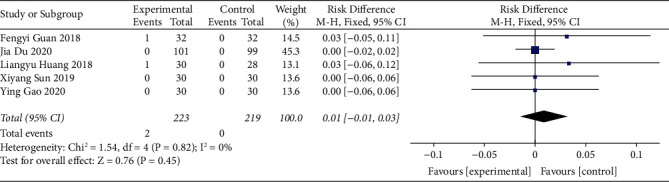
The forest plot of adverse events.

The incidence of adverse events of acupoint catgut embedding for the treatment of poststroke constipation was reported in five studies. As there was no heterogeneity among the included studies (I^2^ = 0% and *P* > 0.10), the fixed-effects model was utilized to combine the data. The results revealed that there was no significant difference in the incidence of adverse events between the two groups. (RD = 0.01, 95% CI (-0.01, 0.03), *P* > 0.05) (Figure 10).

## 4. Sensitivity Analysis

Because of the high heterogeneity among the studies included in the first defecation time, the degree of difficulty in defecation, the stool property score, and PAC-QOL scale score, were eliminated by the one-by-one method to conduct sensitivity analysis. The main source leading to the increase in heterogeneity was not found in the sensitivity analysis of the stool property score, the first defecation time, and the degree of difficulty in defecation. Therefore, the results obtained were relatively stable and reliable. The literature of Jia Du was found to be the main source of increasing heterogeneity in the sensitivity analysis of the PAC-QOL scale score. After excluding this article, the PAC-QOL scale score of the patients in the experimental group that received the catgut embedding therapy was still lower than that of the control group, and the difference between the two groups was statistically significant (*P* < 0.05). Thus, the result obtained was still relatively stable and reliable.

## 5. Publication Bias Analysis

**Figure 11:**
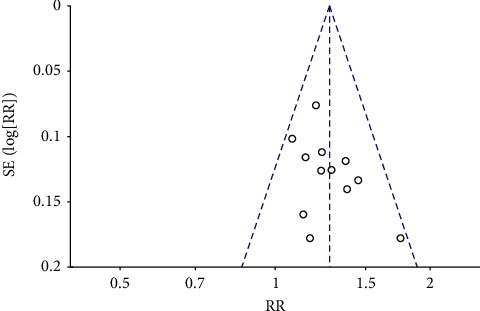
The funnel plot of the efficacy rate.

The efficacy rate of the outcome indices was included in more than 10 studies. The publication bias was evaluated using a funnel chart. Visually, the points on the funnel chart were scattered and not entirely symmetrical, which indicated the possibility of a publication bias (Figure 11). Since the number of studies with first defecation time, defecation sensation score, degree of difficulty in defecation, stool property score, PAC-QOL scale score, symptom integral, and adverse event as outcomes was less than 10, all outcome indicators could not effectively evaluate the publication bias with a funnel chart. Therefore, the Egger test was used to evaluate the publication bias and the results revealed that there was no publication bias (*P* > 0.05).

## 6. Discussion

Constipation, a common complication of stroke, seriously threatens the health of stroke patients. Constipation not only affects the quality of life of patients but also induces various diseases. In severe cases, excessive defecation could increase blood pressure and endanger the health of stroke patients. Therefore, alleviating constipation is essential to improving the quality of life of stroke patients [26]. Although drugs are effective in treating poststroke constipation, people are paying increasing attention to adverse drug reactions.

Acupoint catgut embedding is a novel treatment modality. By implanting modern biomedical materials into the patient's acupoint tissues, the catgut can remain in the body. Thus, the process of catgut embedding can be completed promptly, which forms the long-term stimulatory effect of acupuncture points, realizing the long-term therapy mode. Acupoint embedding is similar in principle to acupuncture and moxibustion but has other advantages. In this therapy, the acupuncture effect is substituted with repeated stimulation of acupoints using implanted thread bodies. The selection of acupoints and the number of thread bodies are determined according to disease severity. Following acupoint catgut embedding, the stimulation of acupoints by thread bodies with movement is similar to acupuncture, which can dredge meridians, regulate viscera, strengthen the body's resistance, and eliminate pathogen. Moreover, the curative effect is stable and lasting [27] Reports show [28] that the mechanism of the acupoint catgut embedding therapy for constipation may stimulate related acupoints and parasympathetic nerves, increasing intestinal peristalsis. This therapy can simultaneously inhibit sympathetic nerves, increasing colorectal fluid secretion, and lubrication.

This systematic review and meta-analysis of the effectiveness and safety of acupoint catgut embedding for the treatment of poststroke constipation have some limitations due to the quality of the literature selected. First, the studies used various acupoints. Second, there is no unified standard for the specific operation of acupoint embedding, such as the embedding method and acupoint selection. Third, the efficacy will also be affected by the operator's technical level, the severity of the patient's condition, and other factors. Fourth, the treatment diversity in the control group of these studies partly affected the consistency of the eligible studies. Finally, the outcome is also affected by factors such as the decision to adopt blinding, the sample size, and the number of centers. Therefore, more rigorously designed, standardized, large-sample, multicenter randomized controlled studies are required to further confirm the results of this study.

## 7. Conclusion

This study demonstrated that acupoint catgut embedding probably has a remarkable curative effect on poststroke constipation. At the same time, it is a treatment method with a definite curative effect, safety, simplicity, and easy acceptance by patients and hence is worthy of clinical application and further research. Nevertheless, more rigorously designed, standardized, large-sample, and multicenter randomized controlled designs are warranted to further verify the findings of this study.

## Figures and Tables

**Table 1:** Basic information of the eligible studies.

First author	Year of publication	Country	Type of study	Interventions	Number of cases			Outcome
				Treatment group	Control group	Treatment group	Control group	
LiLi Zeng [11]	2018	China	RCT	ACE	NACE	30	30	①, ⑦
Huiming Deng [12]	2019	China	RCT	ACE	NACE	29	29	①, ⑤
Yonggang Hao [13]	2020	China	RCT	ACE	NACE	30	30	⑦
Jia Du [14]	2020	China	RCT	ACE	NACE	105	105	④, ⑤, ⑥, ⑧
Zhihong Zou [15]	2014	China	RCT	ACE	NACE	50	50	①
Heyi Yang [16]	2012	China	RCT	ACE	NACE	24	20	①, ②
Wenfeng Wu [17]	2011	China	RCT	ACE	NACE	30	30	⑦
Jinying Guo [18]	2012	China	RCT	ACE	NACE	35	35	①, ②
Zipei Zeng [19]	2012	China	RCT	ACE	NACE	40	40	①
Liangyu Huang [20]	2018	China	RCT	ACE	NACE	30	28	①, ②, ③, ④, ⑥, ⑦, ⑧
Xizong Jin [21]	2016	China	RCT	ACE	NACE	20	20	①, ②, ③, ④, ⑤
Xiyang Sun [22]	2019	China	RCT	ACE	NACE	30	30	①, ②, ③, ④, ⑤, ⑥, ⑦, ⑧
Fengyi Guan [23]	2018	China	RCT	ACE	NACE	32	32	①, ⑥, ⑦, ⑧
Ying Gao [24]	2020	China	RCT	ACE	NACE	30	30	①, ③, ④, ⑤, ⑥, ⑦, ⑧
Guifang Luan [25]	2018	China	RCT	ACE	NACE	30	30	①, ②, ⑦

① Efficacy rate; ② first defecation time; ③ defecation sensation score; ④ degree of difficulty in defecation; ⑤ stool property score; ⑥ PAC-QOL scale score; ⑦ symptom integral; ⑧ adverse event; ACE: acupoint catgut embedding; NACE: nonacupoint catgut embedding.

## Data Availability

The data that support the findings of this study are available from the corresponding authors upon reasonable request.

## Supplementary Materials

Supplementary Materials The PRISMA (Preferred Reporting Items for Systematic Reviews and Meta-Analyses) checklist of this study.
